# Editorial: Insights in women's mental health: 2022

**DOI:** 10.3389/fgwh.2023.1253687

**Published:** 2023-08-02

**Authors:** Jayashri Kulkarni

**Affiliations:** HER Centre Australia, Monash University, Melbourne, VIC, Australia

**Keywords:** trauma, perinatal disorders, COVID, women, mental health

**Editorial on the Research Topic** Insights in women’s mental health: 2022

Why focus on women's mental health? Especially in this era of non-binary, fluid approaches to gender, is a focus on women's mental health relevant or necessary? The answer is a resounding “Yes”!

Women (defined by biological factors and those who identify as women) constitute more than 50% of the population seeking and receiving mental health treatments. Unfortunately, the COVID-19 pandemic and its repercussions has had negative mental health impacts globally, with significantly worse effects on women (1). Women experience more violence, greater poverty, power imbalances and hormone fluctuations leading to specific mental ill health—all of which have led to greater mental ill health (1).

In this Research Topic *Insights in Women's Mental Health: 2022* we have received 4 important articles that reflect women's mental health issues faced across the globe, particularly in the setting of the recent pandemic.

Bishaw et al. performed a multi-centre, cross sectional study in 2020, during the height of the pandemic. They found that the prevalence of generalised anxiety disorder was very high in their study population of 847 Ethiopian pregnant women. Women with fewer than three children, with a high-risk perception of COVID experienced greater generalised anxiety disorder.

Further pregnancy related work is reported in this Research Topic by Prentice et al. In a unique retrospective cohort study, they showed that women with postpartum depression (PPD) are more likely to have adverse childhood experiences compared to the general female population. This finding has important implications regarding the treatment of PPD and prevention of future childhood adverse events.

An important study by Lim et al. described the experiences of social isolation and loneliness experienced by migrant mothers. This Canadian study involving ethno-culturally diverse migrants focussed on women migrants. Multiple vulnerability factors put these women at increased risk of isolation and loneliness with negative health outcomes, negatively affecting the health of their children. While this study was conducted pre-pandemic, there are major implications for the added impact of COVID isolation on migrant mothers and their children.

Ramos eloquently details the many issues that confront Afghan women who are experiencing significant levels of violence due to war related traumas as well as interpersonal violence. Ramos outlines the scarcity of information about Afghan women's suffering and calls for an urgent need to focus on women's trauma experiences to provide better care for them amidst this major humanitarian crisis

More than ever before, a focus on women's mental health is urgently needed, but with new understanding and innovative approaches to meet the increased mental health needs of women. Trauma, as experienced by women in different settings, from early life through to current situations, is a critical central and universal factor underpinning mental ill health. Innovating women's mental health care to meet demands in the post pandemic world, should include a greater integration of factors that traditionally have been regarded as separate domains such as physical health, social and environmental issues as well as psychological coping styles.
